# Comparison of the repeatability and reproducibility of corneal thickness mapping using optical coherence tomography according to tear film break-up time

**DOI:** 10.1186/s12886-024-03536-0

**Published:** 2024-07-05

**Authors:** Kan Lin, Zhiqiang Xu, Hui Wang, Yuzhou Wang, Linzhi Wei, Hongqing Ma, Jian Zhao, Fan Lu, Liang Hu

**Affiliations:** 1 National Clinical Research Center for Ocular Diseases, Eye Hospital, Wenzhou Medical University, Wenzhou, Zhejiang China; 2 National Engineering Research Center of Ophthalmology and Optometry, Eye Hospital, Wenzhou Medical University, Wenzhou, 325027 China; 3 Zhejiang University School of Medicine Second Affiliated Hospital Eye Center, 88 Jiefang Road, Hangzhou, 310009 China; 4 School of Ophthalmology and Optometry, Eye Hospital, Wenzhou Medical University, 270 Xueyuan road, Wenzhou, 325000 Zhejiang China

**Keywords:** Repeatability ande reproducibility, FTBUT, Optical coherence tomography, Corneal thickness mapping, Refractive surgery

## Abstract

**Background:**

To compare the repeatability and reproducibility of corneal and corneal epithelial thickness mapping using anterior segment optical coherence tomography (AS-OCT) according to tear film break-up time (TBUT).

**Methods:**

The included eyes were divided into three subgroups according to TBUT (group 1: TBUT ≤ 5 s, group 2: 5 s < TBUT ≤ 10 s, and group 3: TBUT > 10 s). All eyes were imaged separately thrice by two operators to obtain the thickness maps (TMs) of the cornea and corneal epithelium based on spatial zones encompassing a 9-mm-diameter area. Each TM consisted of 25 areas. Intraoperator (repeatability) and interoperator (reproducibility) standard deviations (Sws), coefficients of variation (CoVs), and intraclass correlation coefficients (ICCs) among the tests were calculated and compared in all the areas.

**Results:**

Altogether, 132 eyes of 67 subjects were included (50, 47, and 35 eyes in groups 1, 2, and 3; respectively). The ICCs of corneal epithelial thickness and corneal thickness were > 0.75 in most of the areas. Pairwise comparisons showed that AS-OCT exhibited lower repeatability in group 1 than in groups 2 and 3 (*P* < 0.05). However groups 2 and 3 showed similar results. Sws and CoVs of corneal epithelial thickness exhibited no significant interoperator differences. While no significant differences were observed in corneal thickness in most of the areas.

**Conclusions:**

TBUT significantly influences the repeatability of corneal and corneal epithelial thickness measurements. Poor tear film stability requires careful evaluation of corneal epithelial thickness.

## Background

Accurate measurement of corneal thickness (CT) and corneal epithelial thickness (ET) plays an important role in corneal refractive surgery [1]. Routine measurement of CT and ET before refractive surgery contributes to the screening of preoperative keratoconus and reduces the incidence of postoperative keratoconus [2–4]. Many previous studies have emphasized the necessity of CT measurement for refractive surgery [5–8]. Recent studies have reported that ET could affect the accuracy of laser ablation [9] in transepithelial photorefractive keratectomy (PRK). In addition, ET should be considered an important factor for the choice of refractive surgery [10]. Furthermore, preoperative ET measurement is helpful in detecting corneal irregularities below the corneal epithelium [11], and postoperative measurement helps in partially explaining the refractive regression [12]. Hence, accurate measurement of CT and ET is vital.

CT and ET can be measured using several modalities including digital ultrasound, corneal topography, and corneal tomography [13–15]. Among these, corneal topography and corneal tomography, which enable thickness mapping of CT and ET in a non-contact manner, are the most widely used modalities in refractive surgery. Anterior segment optical coherence tomography (AS-OCT), a representative modality for corneal tomography, is based on low-coherence interferometry [16]. Compared to corneal topography (mostly Scheimpflug-based devices), AS-OCT has a higher resolution and greater scanning speed in CT measurement. It can also measure the corneal sublayer thickness. It is expected to become a mainstream modality for CT and ET measurements.

However, in AS-OCT examination, the tear film is not identified and directly incorporated into epithelial measurement, since the interface between tear film and corneal epithelium is too small in terms of signal-to-noise ratio to be separated [17]. Lee et al. reported that the repeatability of optical coherence tomography angiography in the retina tended to decrease with a decrease in the stability of tear film [18]. Ruti et al. reported that the repeatability and reproducibility of OCT for ET measurement were significantly lower than those in the normal group [19]. Thus, the quality of the tear film affects OCT measurements in both anterior and posterior ocular segments. Several studies have reported a high prevalence of dry eye disease in patients undergoing corneal refractive surgery [20–22]. In China, 44.62% of the candidates for refractive surgery were considered to have tear film instability (tear film break-up time [TBUT] ≤ 5 s) [23]. It is important to investigate the effects of different tear films on the quality of AS-OCT images.

In the present study, we recruited healthy subjects with normal and short TBUT. We investigated the repeatability and reproducibility of OCT images for CT and ET measurements according to TBUT using the RTVue OCT system (Optovue, Inc.; Fremont, CA, USA), which has shown good repeatability and reproducibility in previous studys [19, 24].

## Materials and methods

### Subjects

The present study was approved by the Institutional Review Committee of Wenzhou Medical University Eye Hospital and was conducted in accordance with the principles of the Declaration of Helsinki. All participants included in this study were candidates for refractive surgery at the Eye Hospital of Wenzhou Medical University between July 2021 and December 2021.

The inclusion criteria were as follows: (1) subjects at least 18 years of age; (2) no history of ocular surgery or trauma; (3) no history of wearing contact lenses or stoppage of wearing contact lenses for at least 14 days without any complications; and (4) no corneal or other ocular pathologies. All examination sequences in this study followed the principle of non-invasive before invasive. All subjects provided written informed consent to participate in the study and underwent all the examinations for both the eyes.

### Tear film break-up time and subgroups

The ocular surface was stained using a fluorescein strip (Jingming, Tianjin, China) wet with one drop of 0.1% sodium hyaluronate eye drops (approximately 50 µL), which was applied to the lower conjunctival sac. The subjects were asked to blink several times. Tear film was observed using a slit-lamp biomicroscope with a cobalt blue filter. The time from the last complete blink to the first tear film break-up (TBUT) was recorded by an ophthalmologist using a stopwatch. The test was repeated thrice, and the average value was calculated. According to TBUT, subjects were divided into three subgroups: group 1, TBUT ≤ 5 s; group 2, 5 s < TBUT ≤ 10 s; and group 3, TBUT > 10 s.

### Optical coherence tomography measurements

Thickness mapping of the cornea (CTM) and corneal epithelium (ETM) was performed using spectral domain OCT (RTVue-XR100, [Optovue, Inc.; Fremont, CA, USA]) equipped with an additional lens (CAM-L module), providing thickness maps (TMs) with a diameter of 9 mm centered on the center of the pupil. RTVue-XR100 achieves a 5-µm depth resolution in the tissues with an 830-nm near-infrared light source. The “Pachymetry Wide” mode was selected before the measurements. The subjects were instructed to blink thrice quickly and watch a red light in front of the eye. The scan started when the “QS” column on the monitor turned green, and the subjects were instructed to maintain the fixation state without blinking during the scan.

**Fig. 1:**
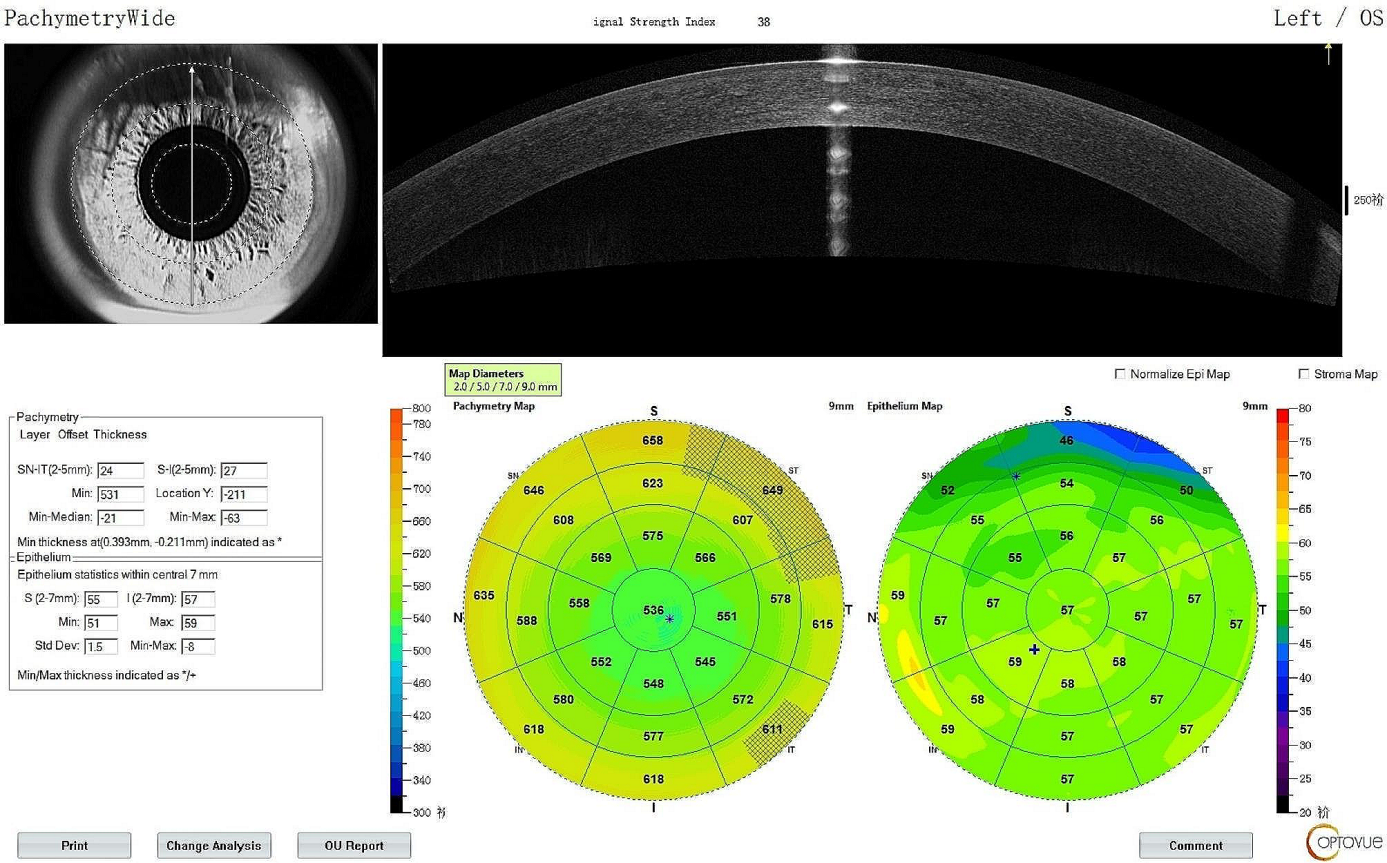
Corneal epithelial thickness and corneal thickness values were obtained in all 25 areas encompassing a 9-mm-diameter zone

Each eye was imaged thrice by two investigators (Kan Lin and Hui Wang). The TMs were obtained in all 25 areas, encompassing a 9-mm-diameter zone in the following four parts (Fig. 1): (1) the corneal center within a diameter of 0–2.0 mm, (2) eight paracentral sectorial areas 2.0–5.0 mm in diameter, (3) eight midperipheral sectorial areas 5.0–7.0 mm in diameter, and (4) eight peripheral sectorial areas 7.0–9.0 mm in diameter. The eight sectorial areas located in the paracentric, midperipheral, and peripheral areas included the superior, superior nasal, nasal, inferior nasal, inferior, inferior temporal, temporal, and superior temporal areas. In addition, the signal strength captured by OCT was recorded.

### Data analysis

A preliminary experiment including ten samples from each group was performed to calculate the sample size. PASS (version 15.0; NCSS, Kaysville, UT, USA) was used in the multiple comparison model, and ensured that this study needed at least 32 samples in each group with a type I error probability set at 0.05 and a type II error probability set at 0.1 (power of 90%). The repeatability (intraoperator) and reproducibility (interoperator) of CTM and ETM in all 25 areas were calculated. Repeatability was represented by the intraclass repeatability coefficient of variation (CoV) of three repeated measurements made by a single operator (Hui Wang) [25]. Reproducibility was represented by the Sw and CoV of the average values calculated from the measurements made by two operators. The Ocular Surface Disease Index (OSDI) scores, age, average ET, average CT, and signal strength were compared among the three groups using one-way analysis of variance. The study incorporated both eyes, hence comparisons of Sw and CoV between the three groups were based on generalized estimating equation comparisons, adjusting for interocular correlations on the statistical results. All analyses were performed using IBM SPSS Statistics version 24.0 (IBM Corp., Armonk, NY, USA).

## Results

Altogether, 132 eyes were included from the 134 eyes of 67 recruited subjects (group 1: 50 eyes, group 2: 47 eyes, and group 3: 35 eyes). Two eyes in group 1 were excluded due to poor OCT image quality. The summary data for each group are presented in Table 1. The mean TBUT values in groups 1, 2, and 3 were 3.63 ± 0.86, 7.10 ± 1.18, and 12.47 ± 1.97 s, respectively; showing a significant difference among the groups (*P* < 0.05). No significant differences were observed in the signal strength of OCT images, OSDI score, or age among the three groups (*P* > 0.05). The CTMs and ETMs exhibited no significant differences among the groups (Table 2).

**Table 1:** Basic Data related to the eyes of the subjects

Group	1 (TBUT < 5)	2 (5 ≤ TBUT < 10)	3 (TBUT ≥ 10)	*P*-value
Included eyes	50	47	35	
TBUT (s)	3.63 ± 0.86 (range 1.91–4.98)	7.10 ± 1.18 (range 5.03–9.83)	12.47 ± 1.97 (range 10.00-17.73)	**< 0.001** ^***^
Male/Female	15/35	28/19	28/7	**< 0.001** ^***^
Age (years)	25.26 ± 5.50 (range 17–34)	25.32 ± 5.83 (range 17–35)	22.57 ± 6.21 (range 17–35)	0.680
Signal strength	35.57 ± 3.03 (range 29.33-41.00)	35.06 ± 2.32 (range 29.67-39.00)	35.14 ± 2.15 (range 31.33–39.33)	0.600
OSDI score	10.70 ± 14.73 (range 0-70.83)	6.51 ± 6.05 (range 0-20.83)	8.92 ± 8.85 (range 0-35.42)	0.168

TBUT: tear film break-up time, OSDI: Ocular Surface Disease Index

***Statistically significant(*p* < 0.001), Data are presented as absolute numbers or means ± standard deviations

**Table 2:** Average corneal epithelial thickness and corneal thickness in all 25 areas of all subjects

Zone	ET				CT			
	TBUT < 5	5 ≤ TBUT < 10	TBUT ≥ 10	*P*-value	TBUT < 5	5 ≤ TBUT < 10	TBUT ≥ 10	*P*-value
C	54.35 ± 3.75	54.51 ± 2.50	55.75 ± 6.47	0.138	540.25 ± 31.96	544.28 ± 34.53	542.41 ± 24.37	0.820
s25	53.33 ± 3.89	53.69 ± 2.52	54.96 ± 6.34	0.083	585.99 ± 33.98	587.59 ± 36.94	588.20 ± 29.11	0.952
s57	50.38 ± 4.28	51.62 ± 3.46	51.83 ± 6.30	0.164	636.67 ± 34.13	635.92 ± 37.37	637.00 ± 32.08	0.990
s79	45.35 ± 4.30	46.50 ± 3.54	46.33 ± 5.71	0.281	683.99 ± 39.66	682.84 ± 38.29	679.98 ± 37.02	0.894
sn25	54.04 ± 3.86	53.94 ± 2.39	55.57 ± 6.35	0.053	583.63 ± 34.73	585.45 ± 36.36	584.27 ± 28.14	0.996
sn57	52.69 ± 4.01	53.20 ± 2.97	54.18 ± 6.34	0.164	632.89 ± 36.56	632.79 ± 38.83	630.41 ± 30.95	0.944
sn79	48.76 ± 3.92	49.59 ± 3.79	49.89 ± 6.36	0.396	683.81 ± 40.67	683.35 ± 45.82	677.30 ± 33.16	0.742
n25	54.89 ± 3.81	54.65 ± 2.56	56.21 ± 6.52	0.101	572.85 ± 34.85	575.03 ± 35.05	572.47 ± 26.82	0.927
n57	55.01 ± 3.89	54.75 ± 2.37	55.93 ± 6.38	0.253	614.40 ± 38.28	616.24 ± 36.73	611.96 ± 30.47	0.869
n79	55.12 ± 3.61	54.99 ± 2.58	55.16 ± 6.33	0.964	663.97 ± 40.85	666.82 ± 40.65	660.42 ± 34.21	0.769
in25	55.63 ± 4.09	55.36 ± 2.81	56.88 ± 6.67	0.157	563.28 ± 33.51	567.04 ± 34.54	563.48 ± 26.11	0.823
in57	55.55 ± 3.67	55.57 ± 2.58	56.41 ± 6.46	0.415	602.28 ± 35.27	604.53 ± 37.24	601.33 ± 29.77	0.911
in79	54.36 ± 3.22	54.67 ± 2.53	54.95 ± 6.30	0.664	649.60 ± 39.27	652.20 ± 41.39	648.48 ± 34.86	0.905
i25	55.59 ± 4.03	55.52 ± 2.84	56.94 ± 6.69	0.160	555.23 ± 32.13	559.30 ± 35.48	557.32 ± 25.64	0.824
i57	55.12 ± 3.43	55.09 ± 2.53	56.25 ± 6.42	0.179	592.21 ± 32.64	594.06 ± 38.42	593.34 ± 29.60	0.965
i79	52.97 ± 3.52	53.48 ± 2.83	54.50 ± 6.49	0.137	635.92 ± 36.45	636.00 ± 41.88	635.03 ± 32.98	0.992
it25	54.89 ± 3.88	55.14 ± 2.73	56.37 ± 6.62	0.150	550.25 ± 32.08	555.13 ± 35.70	553.51 ± 24.91	0.748
it57	54.62 ± 3.37	54.63 ± 2.67	56.01 ± 6.45	0.090	583.04 ± 33.75	586.45 ± 39.11	585.74 ± 28.87	0.881
it79	53.43 ± 3.20	53.89 ± 2.47	54.87 ± 6.21	0.081	628.35 ± 38.66	628.76 ± 45.22	627.73 ± 34.00	0.994
t25	54.28 ± 3.64	54.54 ± 2.52	55.67 ± 6.49	0.156	554.63 ± 32.14	559.55 ± 35.79	558.29 ± 25.51	0.741
t57	53.75 ± 3.31	54.21 ± 2.53	55.39 ± 6.38	0.057	585.78 ± 34.17	591.03 ± 37.59	589.04 ± 29.12	0.753
t79	52.58 ± 3.24	53.35 ± 2.36	54.05 ± 6.09	0.064	627.95 ± 37.09	633.63 ± 40.80	629.77 ± 34.29	0.760
st25	53.75 ± 3.76	54.09 ± 2.46	55.17 ± 6.39	0.144	571.41 ± 33.27	574.67 ± 36.94	574.81 ± 28.09	0.861
st57	52.50 ± 3.75	53.16 ± 2.71	53.74 ± 6.25	0.236	613.34 ± 35.01	615.07 ± 38.45	616.90 ± 32.30	0.904
st79	48.90 ± 3.90	50.11 ± 2.91	49.77 ± 5.89	0.201	658.43 ± 36.92	657.08 ± 39.86	661.50 ± 37.53	0.936

ET: corneal epithelial thickness, CT: corneal thickness, TBUT: tear film break-up time, C: central, S: superior, SN: superior nasal, N: nasal, IN: inferior nasal, I: inferior, IT: inferior temporal, T: temporal, ST: superior temporal

Data are presented as absolute numbers or means ± standard deviations

The ICCs of single-surveyor measurements (Hui Wang) are presented in Table 3. CT exhibited greater ICC values than ET. ICC values were > 0.75 in most of the areas and never < 0.4 in any of the areas. The ICC of ETM was > 0.75 in group 3.

**Table 3:** The ICC of corneal epithelial thickness and corneal thickness measured by a surveyor three times

Zone	ET			CT		
	TBUT < 5	5 ≤ TBUT<10	TBUT ≥ 10	TBUT < 5	5 ≤ TBUT<10	TBUT ≥ 10
C	0.77	**0.69**	0.94	0.99	0.99	0.99
s25	0.78	**0.70**	0.91	0.94	0.98	0.95
s57	**0.71**	0.82	0.81	0.87	0.97	0.93
s79	**0.59**	**0.70**	0.52	0.80	0.94	0.91
sn25	0.80	**0.66**	0.94	0.95	0.97	0.95
sn57	0.81	0.79	0.86	0.90	0.87	0.93
sn79	0.77	0.84	0.78	0.82	**0.60**	0.89
n25	0.79	0.79	0.92	0.95	0.96	0.96
n57	0.83	0.83	0.92	0.92	0.90	0.92
n79	0.88	0.86	0.92	0.86	**0.71**	0.85
in25	0.78	**0.69**	0.93	0.96	0.91	0.96
in57	0.80	0.80	0.92	0.93	0.94	0.90
in79	0.83	0.86	0.95	0.86	0.83	**0.70**
i25	0.77	**0.73**	0.92	0.97	0.99	0.97
i57	0.75	0.79	0.92	0.92	0.97	0.94
i79	**0.48**	0.84	0.96	0.77	0.95	0.92
it25	0.79	**0.73**	0.95	0.98	0.99	0.98
it57	0.75	**0.73**	0.94	0.85	0.94	0.92
it79	0.86	**0.73**	0.95	**0.53**	0.78	0.80
t25	**0.74**	0.78	0.94	0.98	0.99	0.98
t57	**0.72**	**0.74**	0.92	0.94	0.94	0.95
t79	0.84	0.77	0.91	0.86	0.78	0.89
st25	0.76	0.82	0.93	0.96	0.98	0.96
st57	0.78	0.82	0.87	0.93	0.97	0.90
st79	0.75	0.78	0.77	0.87	0.93	**0.67**

C: central; S: superior; SN: superior nasal; N: nasal; IN: inferior nasal; I: inferior; IT: inferior temporal; T: temporal; ST: superior temporal; ET: corneal epithelial thickness; CT: corneal thickness

**Fig. 2:**
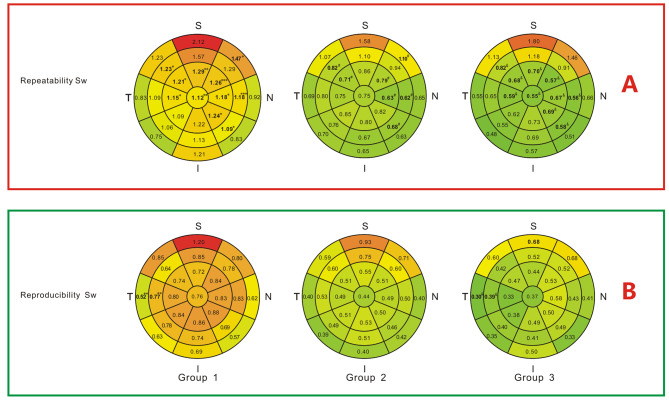
Repeatability and reproducibility standard deviations (Sws) of corneal epithelial thickness in 25 areas. * Significant difference among the three groups(*p* < 0.05), **p < 0.01, ***p < 0.001. ^#^ Significant difference between group 1 and group 2 in pairwise comparisons. ^&^ Significant difference between group 1 and group 3 in pairwise comparisons

**Fig. 3:**
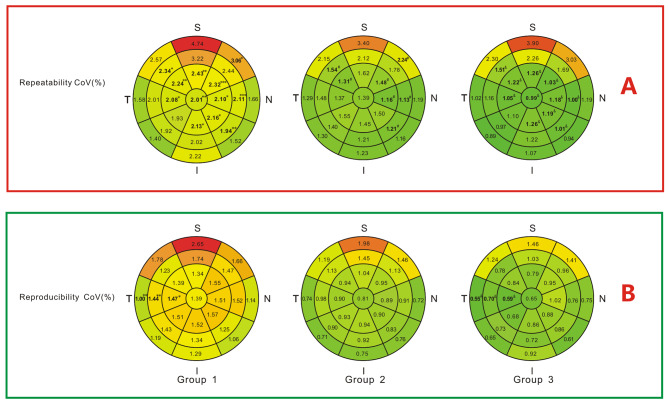
Repeatability and reproducibility coefficients of variation (CoVs) of corneal epithelial thickness in 25 areas. * Significant difference among the three groups(*p* < 0.05), **p < 0.01, ***p < 0.001. ^#^ Significant difference between group 1 and group 2 in pairwise comparisons. ^&^ Significant difference between group 1 and group 3 in pairwise comparisons

Intraoperator Sw and CoV of ETM showed significant differences among the groups in several areas (Figs. 2A and 3A). These values decreased with an increase in TBUT. Eleven areas showed statistically significant differences in Sw, while 12 areas showed statistically significant differences in CoV among the groups. Interoperator Sw and CoV of ETM for all 25 areas are shown in Figs. 2B and 3B. Altogether, only 2 areas showed statistically significant differences in Sw, while 3 areas showed statistically significant differences in CoV among the three groups. Pairwise comparisons showed that in all the areas, no significant differences were observed between group 2 and group 3 (*P* > 0.05).

**Fig. 4:**
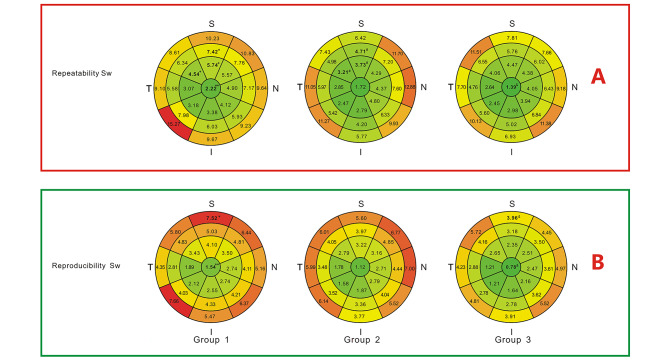
Repeatability and reproducibility standard deviations (Sws) of corneal thickness in 25 areas. * Significant difference among the three groups(*p* < 0.05), **p < 0.01, ***p < 0.001. ^#^ Significant difference between group 1 and group 2 in pairwise comparisons. ^&^ Significant difference between group 1 and group 3 in pairwise comparisons

**Fig. 5:**
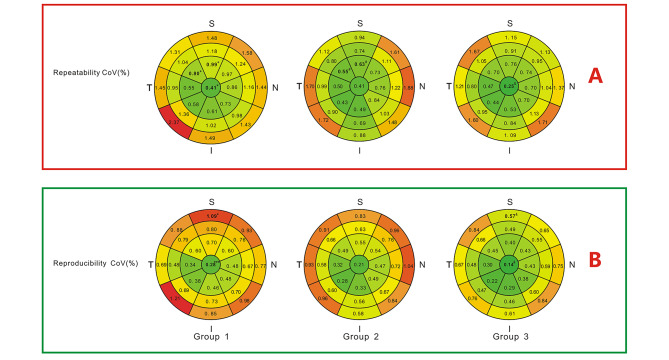
Repeatability and reproducibility coefficients of variation (CoVs) of corneal thickness in 25 areas. * Significant difference among the three groups(*p* < 0.05), **p < 0.01, ***p < 0.001. ^#^ Significant difference between group 1 and group 2 in pairwise comparisons. ^&^ Significant difference between group 1 and group 3 in pairwise comparisons

Intraoperator Sw and CoV of CTM in most of the areas showed no significant differences among the three groups (Figs. 4 and 5) except only two and three areas respectively(*P* < 0.05). Interoperator Sw and CoV analyses were also performed similarly. In both intraoperator and interoperator analyses, a few areas showed significant differences among the three groups. It is remarkable that groups 2 and 3 exhibited no significant differences in the repeatability and reproducibility of ETM or CTM.

## Discussion

In the present study, CT and ET in 25 areas encompassing a 9-mm-diameter zone were measured in subjects with different TBUT values using RTVue AS-OCT system. Repeatability and reproducibility were calculated and compared. The main results were as follows.

1. Repeatability of ET were significantly lower in the TBUT ≤ 5 s group (group 1) compared to the remaining groups in quite a large area.
2. Differences were found in only a few areas between group 2 and group 3.
3. A few areas showed significant differences of CTM intraoperator or interoperator.

In the present study, ETM in group 1 exhibited lower repeatability than that in the remaining two groups. However, the repeatability in groups 2 and 3 were similar. Sella et al. reported that the repeatability of ET measurement using OCT was significantly lower in subjects with dry eyes than in normal subjects [19]. This finding is consistent with our results, indicating that a shorter TBUT is associated with lower repeatability. Tear break-up may occurs in the eye during the entire measurement process, which caused repeatability and reproducibility decline. Ma et al. reported that in subjects with dry eyes, contact lens wearers, subjects with keratoconus, and subjects who have undergone laser-assisted in situ keratomileusis (LASIK) or PRK; the repeatability of ET measurement using OCT was worse than that in normal subjects [24]. In their study, all aforementioned subgroups were characterized by instability of the ocular surface microenvironment and tear film. This finding is consistent with our hypothesis. On the other hand, few significant differences were found in the reproducibility of ETM measurements among the three groups in this study. It suggests that the reproducibility of OCT measurements of ET performed well after repeated measurements.

Reportedly OCT exhibited similar repeatability and reproducibility of CT measurement in eyes with contact lenses, dry eyes, and eyes that have undergone LASIK/PRK [24]. However, the ocular surface microenvironment obviously differed between these eyes and normal eyes. In the present study, both repeatability and reproducibility of CT showed little differences among the groups when compared with differences in ET. TBUT mainly reflects tear film stability on the corneal surface. Unstable tear film leads to a short TBUT, which affects the accuracy of the measurement. However, the tear film is extremely thin (2–5.5 µ) [26–29]. Hence, the effect is very small for relatively larger measurements such as measurement of CT.

In the analysis of ICCs in all the areas, AS-OCT was accurate regardless of TBUT. However, ICC was still < 0.75 in several areas concentrated near the nasal and superior sides. Since our study subjects were of Asian ethnicity and included subjects with varying degrees of epicanthus, these were the areas most severely affected by epicanthus [30]. Even when the eyes are exposed as much as possible, the nasal and superior sides are easily obscured by the shadows of the eyelids and conjunctiva, affecting the accuracy of the measurements.

The present study was designed to compare the repeatability and reproducibility of corneal and corneal epithelial TMs generated by AS-OCT according to TBUT. CT and ET measurements are important ocular examinations for refractive surgery [31]. The majority of the candidates for refractive surgery, especially those in China, suffer from dry eye disease or unstable tear film. Thus, it is particularly important to determine whether CT and ET measurements can accurately reflect the real situation of subjects having different TBUT values [20–23]. This study evaluated candidates for refractive surgery with different TBUT values, and the results showed that AS-OCT had lower repeatability and reproducibility of ET measurement in subjects with TBUT < 5s than in those from other TBUT subgroups. It may provide information relevant to clinical refractive surgery when ET measurements are unreliable.

In our study, despite the 5 μm resolution of commercial OCT systems, which leads to unavoidable measurement deviations, we observed increased intragroup variability and decreased reproducibility of ET measurements as TBUT decreased. This suggests that while the resolution limits do introduce error, tear film instability significantly impacts ET measurement variability. Therefore, both technical limitations and biological variability should be considered when interpreting ET measurements in the context of poor tear film stability.

In this study, repeatability values are better than reproducibility values. However, the repeatability was calculated from the data of three repeated measurements made by a single operator. The reproducibility was calculated from the data of average values measurements made by two operators. This means that a part of random errors have been excluded from the reproducibility comparison.

This study has some limitations. (1) Both the eyes of the subjects were included in the study. (2) The age of the subjects was significantly different among the three TBUT subgroups. However, repeatability and reproducibility analyzing between the three groups in this study were compared by generalized estimating equations adjusted for interocular correlation and gender correlation, hence this had less impact on the findings of this study. ETM has been widely used in many clinical studies on bullous keratopathy and studies involving postoperative evaluation of keratoconus crosslinking, pterygium, and granular corneal dystrophy [32–36]. Further studies are needed to investigate the effect of errors in epithelial measurements caused by the tear film on these medical conditions.

## Conclusions

In conclusion, AS-OCT provided good repeatability and reproducibility of CT and ET measurements in all TBUT subgroups. TBUT influences the repeatability of CT and ET measurements. Poor tear film stability requires careful evaluation of ET.

## Data Availability

The datasets used and/or analysed during the current study are available from the corresponding author on reasonable request.

## Abbreviations

AS-OCT: Anterior segment optical coherence tomography
TBUT: Tear film break-up time
TMs: Thickness maps
Sws: Standard deviations
CoVs: Coefficients of variation
ICCs: Intraclass correlation coefficients
CT: Corneal thickness
ET: Epithelial thickness
PRK: Photorefractive keratectomy
CTM: Thickness mapping of the cornea
ETM: Corneal epithelium
OSDI: Ocular Surface Disease Index

## References

[1] Salomão M, Hofling-Lima A, Lopes B, Canedo A, Dawson D, Carneiro-Freitas R, Ambrósio R (2017). Role of the corneal epithelium measurements in keratorefractive surgery. Curr Opin Ophthalmol.

[2] Xu Z, Jiang J, Yang C, Huang S, Peng M, Li W, Cui L, Wang J, Lu F, Shen M (2016). Value of corneal epithelial and Bowman’s layer vertical thickness profiles generated by UHR-OCT for sub-clinical keratoconus diagnosis. Sci Rep.

[3] Wu S, Tao A, Jiang H, Xu Z, Perez V, Wang J (2014). Vertical and horizontal corneal epithelial thickness profile using ultra-high resolution and long scan depth optical coherence tomography. PLoS ONE.

[4] Kanellopoulos AJ, Asimellis G (2014). OCT corneal epithelial topographic asymmetry as a sensitive diagnostic tool for early and advancing keratoconus. Clin Ophthalmol (Auckland NZ).

[5] Maldonado M, Ruiz-Oblitas L, Munuera J, Aliseda D, García-Layana A, Moreno-Montañés J (2000). Optical coherence tomography evaluation of the corneal cap and stromal bed features after laser in situ keratomileusis for high myopia and astigmatism. Ophthalmology.

[6] Schuh A, Kolb C, Mayer W, Vounotrypidis E, Kreutzer T, Kohnen T, Priglinger S, Shajari M, Kook D (2021). Comparison of changes in corneal volume and corneal thickness after myopia correction between LASIK and SMILE. PLoS ONE.

[7] Shah S, Laiquzzaman M (2009). Comparison of corneal biomechanics in pre and post-refractive surgery and keratoconic eyes by Ocular Response Analyser. Contact lens Anterior eye: J Br Contact Lens Association.

[8] Matsuda J, Hieda O, Kinoshita S (2008). Comparison of central corneal thickness measurements by Orbscan II and Pentacam after corneal refractive surgery. Jpn J Ophthalmol.

[9] Jun J, Kang DSY, Arba-Mosquera S, Kim EK, Seo KY, Kim TI (2018). Clinical outcomes of Transepithelial Photorefractive Keratectomy according to epithelial thickness. J Refractive Surg (Thorofare NJ: 1995).

[10] Asroui L, Dupps W, Randleman J (2022). Determining the utility of epithelial thickness mapping in refractive surgery evaluations. Am J Ophthalmol.

[11] Reinstein DZ, Archer TJ, Gobbe M (2012). Refractive and topographic errors in topography-guided ablation produced by epithelial compensation predicted by 3D Artemis VHF digital ultrasound stromal and epithelial thickness mapping. J Refractive Surg (Thorofare NJ: 1995).

[12] Pokroy R, Mimouni M, Sela T, Munzer G, Kaiserman I (2016). Myopic laser in situ keratomileusis retreatment: incidence and associations. J Cataract Refract Surg.

[13] Williams R, Fink B, King-Smith P, Mitchell G (2011). Central corneal thickness measurements: using an ultrasonic instrument and 4 optical instruments. Cornea.

[14] Bayhan H, Aslan Bayhan S, Can I (2014). Comparison of central corneal thickness measurements with three new optical devices and a standard ultrasonic pachymeter. Int J Ophthalmol.

[15] Yap T, Archer T, Gobbe M, Reinstein D (2016). Comparison of central corneal thickness between Fourier-Domain OCT, very high-frequency Digital Ultrasound, and Scheimpflug Imaging Systems. J Refractive Surg (Thorofare NJ: 1995).

[16] Huang D, Swanson EA, Lin CP, Schuman JS, Stinson WG, Chang W, Hee MR, Flotte T, Gregory K, Puliafito CA (1991). Optical coherence tomography. Sci (New York NY).

[17] Hwang E, Schallhorn J, Randleman J (2020). Utility of regional epithelial thickness measurements in corneal evaluations. Surv Ophthalmol.

[18] Lee WH, Lim HB, Kim J, Ryu CK, Shin YI, Kim JY (2021). Repeatability of Macular Microvasculature measurements using Optical Coherence Tomography Angiography according to tear Breakup Time in Dry Eye Disease. Retina.

[19] Sella R, Zangwill L, Weinreb R, Afshari N (2019). Repeatability and reproducibility of corneal epithelial thickness mapping with spectral-domain optical coherence tomography in normal and diseased cornea eyes. Am J Ophthalmol.

[20] Salomão MQ, Ambrósio R, Wilson SE (2009). Dry eye associated with laser in situ keratomileusis: mechanical microkeratome versus femtosecond laser. J Cataract Refract Surg.

[21] Mian SI, Li AY, Dutta S, Musch DC, Shtein RM (2009). Dry eyes and corneal sensation after laser in situ keratomileusis with femtosecond laser flap creation effect of hinge position, hinge angle, and flap thickness. J Cataract Refract Surg.

[22] Hammond MD, Madigan WP, Bower KS (2005). Refractive surgery in the United States Army, 2000–2003. Ophthalmology.

[23] Li M, Zeng L, Mi S, Li Y, Liu Z, Yu K, Hu Q, Li H, Ma D, Zhou Y (2021). A Multicenter Study of the prevalence of Dry Eye Disease in Chinese refractive surgery candidates. Ophthalmic Res.

[24] Ma J, Wang L, Weikert M, Montes de Oca I, Koch D (2019). Evaluation of the repeatability and reproducibility of corneal epithelial thickness mapping for a 9-mm zone using Optical Coherence Tomography. Cornea.

[25] McAlinden C, Khadka J, Pesudovs K (2015). Precision (repeatability and reproducibility) studies and sample-size calculation. J Cataract Refract Surg.

[26] Werkmeister RM, Alex A, Kaya S, Unterhuber A, Hofer B, Riedl J, Bronhagl M, Vietauer M, Schmidl D, Schmoll T (2013). Measurement of tear film thickness using ultrahigh-resolution optical coherence tomography. Investig Ophthalmol Vis Sci.

[27] King-Smith PE, Fink BA, Fogt N, Nichols KK, Hill RM, Wilson GS (2000). The thickness of the human precorneal tear film: evidence from reflection spectra. Invest Ophthalmol Vis Sci.

[28] Wang J, Fonn D, Simpson TL, Jones L (2003). Precorneal and pre- and postlens tear film thickness measured indirectly with optical coherence tomography. Invest Ophthalmol Vis Sci.

[29] Schmoll T, Unterhuber A, Kolbitsch C, Le T, Stingl A, Leitgeb R (2012). Precise thickness measurements of Bowman’s layer, epithelium, and tear film. Optom Vis Sci.

[30] Johnson CC (1968). Epicanthus. Am J Ophthalmol.

[31] Fogla R, Luthra G, Chhabra A, Gupta K, Dalal R, Khamar P (2020). Preferred practice patterns for photorefractive keratectomy surgery. Indian J Ophthalmol.

[32] Rocha KM, Perez-Straziota CE, Stulting RD, Randleman JB (2014). Epithelial and stromal remodeling after corneal collagen cross-linking evaluated by spectral-domain OCT. J Refractive Surg (Thorofare NJ: 1995).

[33] Kasai K, Kato N, Den S, Konomi K, Shinzawa M, Shimazaki J (2019). A prospective, randomized clinical study comparing accelerated corneal collagen crosslinking with 5% NaCl hypertonic saline for bullous keratopathy in Asian eyes. Medicine.

[34] Kamiya K, Takahashi M, Shoji N. Effect of Platelet-Rich Plasma on Corneal Epithelial Healing after Phototherapeutic Keratectomy: An Intraindividual Contralateral Randomized Study. *BioMed research international* 2021, 2021:5752248. 10.1155/2021/5752248 PMC8643227 34873572

[35] Chaidaroon W, Satayawut N, Tananuvat N (2021). Effect of 2% hyaluronic acid on the rate of Healing of corneal epithelial defect after pterygium surgery: a Randomized Controlled Trial. Drug Des Devel Ther.

[36] Haberman ID, Lang PZ, Broncano AF, Kim SW, Hafezi F, Randleman JB (2018). Epithelial remodeling after corneal crosslinking using higher fluence and accelerated treatment time. J Cataract Refract Surg.

